# SBA-Pr-Is-TAP Functionalized Nanostructured Silica as a Highly Selective Fluorescent Chemosensor for Fe^3+^ and Cr_2_O_7_^2−^ Ions in Aqueous Media

**DOI:** 10.3390/nano11102533

**Published:** 2021-09-28

**Authors:** Ghodsi Mohammadi Ziarani, Maryam Akhgar, Fatemeh Mohajer, Alireza Badiei, Rafael Luque

**Affiliations:** 1 Department of Chemistry, Faculty of Physics and Chemistry, Alzahra University, Tehran 1993893979, Iran; m.akhgar@student.alzahra.ac.ir (M.A.); f.mohajer@alzahra.ac.ir (F.M.); 2 School of Chemistry, College of Science, University of Tehran, Tehran 1417614411, Iran; abadiei@khayam.ut.ac.ir; 3 Departamento de Quimica Organica, Grupo FQM-383, Campus de Rabanales, Universidad de Cordoba, Edificio Marie Curie, Ctra Nnal IV-A, Km 396, E14014 Córdoba, Spain

**Keywords:** SBA-Pr-Is-TAP, functionalized mesoporous silica, chemosensor, fluorescent spectroscopy, aqueous media, Fe^3+^ and Cr_2_O_7_^2−^ ions, 2,4,6-triaminoprymidine, isatin

## Abstract

SBA-Pr-Is-TAP was synthesized via functionalization of SBA-15. The synthesized hybrid nanomaterial was characterized by various techniques including FT-IR, TGA, XRD, SEM, and BET. SBA-Pr-Is-TAP could precisely bind Fe^3+^ and Cr_2_O_7_^2−^ ions among a range of different species in aqueous media, consequently acting as a nanoporous chemosensor of Fe^3+^ and Cr_2_O_7_^2−^ ions. An excellent linear relation was observed between the nanoporous chemosensor and ion concentrations, with acceptable detection limits of 2.43 × 10^−6^ M and 3.96 × 10^−7^ M for Fe^3+^ and Cr_2_O_7_^2−^ ions respectively.

## 1. Introduction

Ordered mesoporous silica was discovered for the first time in 1992 [1]. Several mesoporous scaffolds have been classified into three types based on their pore forms: round cage, cylindrical, and continuous channel [2]. Properties including morphology, pore size, and applications have been changed and improved by functionalization reactions [3,4,5]. The most popular form of mesoporous nanosilicas is SBA-15, which has attracted significant consideration due to its benefits [6,7,8,9,10,11,12,13]. SBA-15 nanoporous silica has excellent properties including adjustable cavities, large surface area, thick framework walls, complementary porosity, and thermal stability [14,15,16].

Functionalized SBA-15 by different organic compounds can provide access to different chemosensors to detect different ions. For example, a highly ordered mesoporous silica material (SBA-15) functionalized with 5-(4-carboxy-phenylazo)-8-hydroxyquinoline (CPA-8-HQL) acts as a fluorescence chemosensor for sensing Pb^2+^ ions [17]. Furthermore, another fluorescence nano-chemosensor for Cr_2_O_7_^2−^ anion was synthesized by the assembly of a fluorescent aluminum complex of 8-hydroxyquinoline (AlQ*_x_*) within the channels of modified SBA-15 [18].

Since isatin and its derivatives have excellent properties including biological and pharmacological activities, these have been used in various organic syntheses [19,20,21]. Functionalized nanoporous materials by Fe^3+^ ion can play a vital role in important processes including the transformation of an electron in DNA and RNA as well as biological metabolism [22]. The lack or excess of iron has been reported to lead to a number of diseases such as skin problems, iron shortage anemia (IDA), low immunity, Alzheimer’s, Huntington’s, and Parkinson’s [23]. The increase of iron could disturb biological systems and cause water contamination [24]. On the other hand, Cr_2_O_7_^2−^ has also been made by industrial companies as one of the heavy-metal water pollutants. Consequently, low-cost, sensitive, selective, and fast detection of Fe^3+^ and Cr_2_O_7_^2−^ will be highly valuable [25]. In continuation with recent research from our groups [26,27,28,29,30,31,32,33,34,35], the design of a nanostructured chemosensor based on modified SBA-15 (SBA-Pr-Is-TAP) was attempted in this work, with its chemosensor activities studied in the detection of Fe^3+^ and Cr_2_O_7_^2−^ in the presence of other ions.

## 2. Results and Discussion

**Scheme 1:**
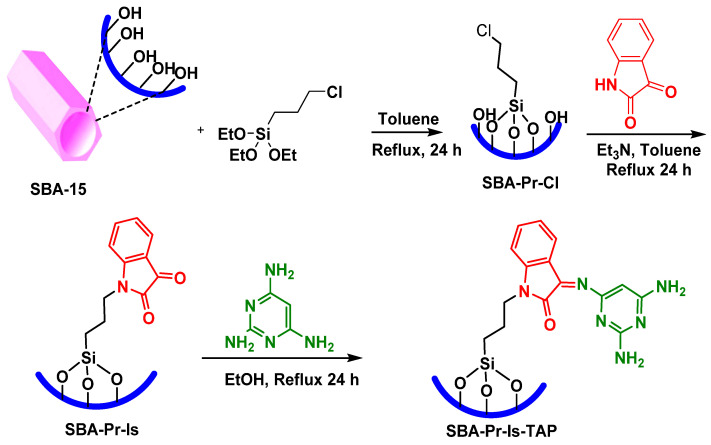
Schematic preparation of SBA-Pr-Is-TAP.

The overall process for the preparation of the SBA-Pr-Is-TAP is illustrated in Scheme 1. Firstly, SBA-15 was functionalized with (3-chlorpropyl) triethoxysilane to provide SBA-Pr-Cl, which was modified by isatin to give SBA-Pr-Is. In the final step, SBA-Pr-Is were reacted with 2,4,6-triaminopyrimidine to provide SBA-Pr-Is-TAP, fully characterized by different analysis methods including IR, TGA, XRD, etc. Full information on characterization equipment and experiments is provided in the experimental section.

Three amine groups on 2,4,6-triaminopyrimidine can potentially react with carbonyl groups for the formation of a Schiff Base. NH_2_ groups in positions of 4 and 6 are chemically equivalent, therefore two types of amine groups present for Schiff base reaction (two amine groups at positions of 4 and 6, and the other at position 2). On the other hand, the basicity of the amino group at position 2 is lower than the others, suggesting that the surface adduct should be formed via reaction at the 4 or 6 positions.

**Figure 1:**
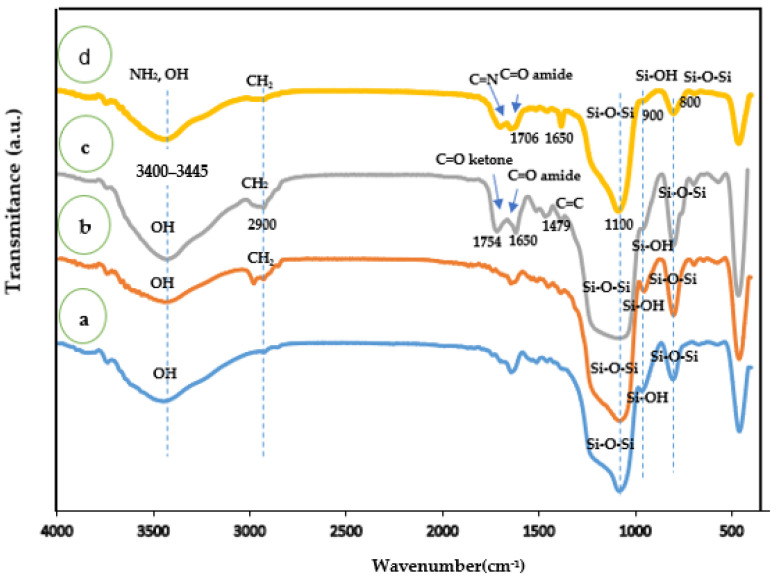
FT-IR spectra of (**a**) SBA-15, (**b**) SBA-Pr-Cl, (**c**) SBA-Pr-Is, and (**d**) SBA-Pr-Is-TAP.

FT-IR spectroscopy shows the organic moieties covalently bonded on the SBA-15 surface. FT-IR spectra for SBA-15, SBA-Pr-Cl, SBA-Pr-Is, and SBA-Pr-Is-TAP are depicted in Figure 1. The bands at 800 and 1100 cm^−1^, respectively, are related to Si-O-Si bonds, while the band at 960 is related to Si-OH. Then, the band around 3456 cm^−1^ could be attributed to O–H stretching of Si-OH functional groups. The peak around 1650 cm^−1^ is associated with C=O stretching vibration and the bending vibrations of water molecules (in spectrum c). The presence of a new band in spectra b and c in at 2881–2990 cm^−1^ range is due to the stretching vibrations of methylene in propyl groups. In c, the new band at 1479 and 1754 cm^−1^ corresponded to C=C and C=O stretching vibrations. The new band at 1706 is related to C=N groups. In spectra d, the new band at 3400 cm^−1^ is related to NH_2_ groups of triaminopyrimidine. These new bands proved that aliphatic compounds, isatin, and triaminopyrimidine were successfully immobilized on the surface of SBA-15.

**Figure 2:**
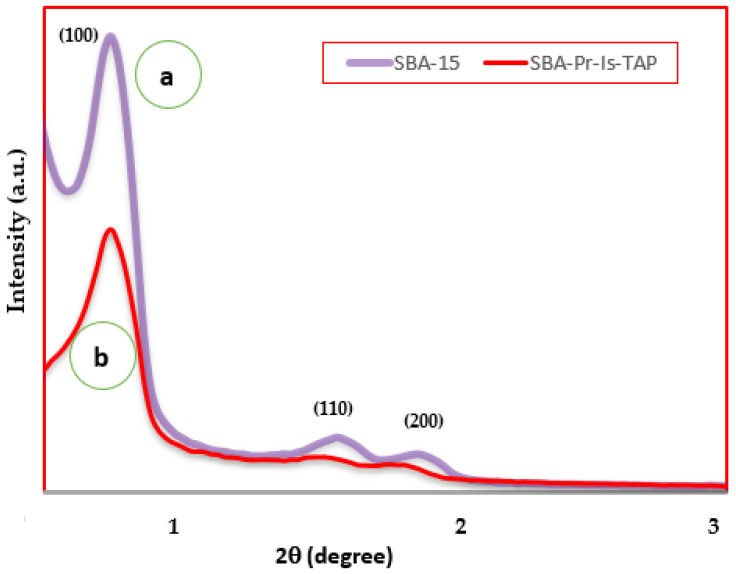
Low-angle XRD profiles of (**a**) SBA-15 and (**b**) SBA-Pr-Is-TAP.

X-ray powder diffraction (XRD) of SBA-15 and SBA-Pr-Is-TAP is shown in Figure 2. According to the obtained results, a strong reflection was observed at around 2θ = 1° corresponding to (100) plane, while the other two intense reflections at ca. 2θ = 1.8° and 2.0° could be assigned to (110) and (200) planes. These reflections demonstrate that the functionalized products maintained highly ordered mesoporous hexagonal structures. Comparing the XRD of SBA-15 with SBA-Pr-Is and SBA-Pr-Is-TAP, the same diffraction lines could be visualized in all three materials (with different intensities). These results confirmed the successful incorporation of the organic moieties onto SBA-15.

**Figure 3:**
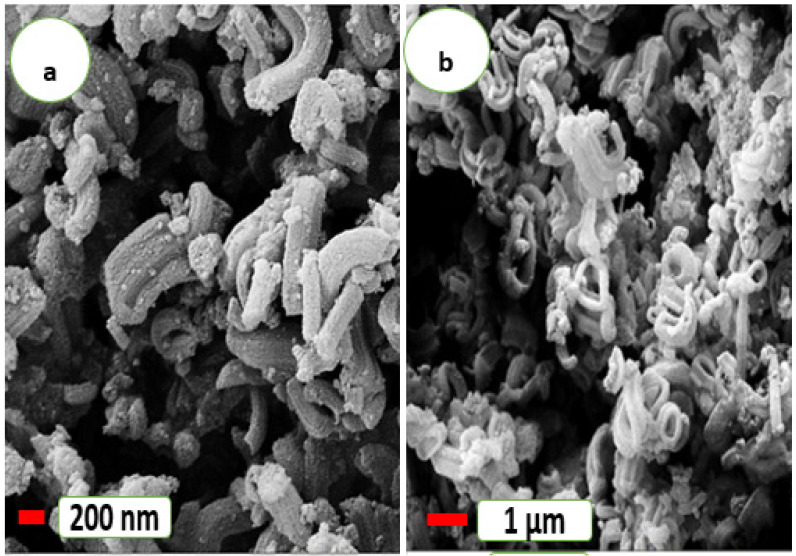
SEM images of (**a**) SBA-15 and (**b**) SBA-Pr-Is-TAP.

**Figure 4:**
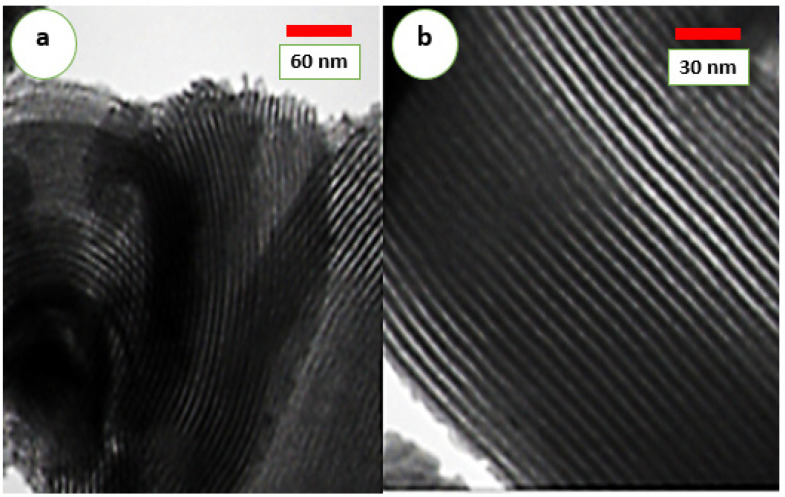
TEM images of (**a**) SBA-15 and (**b**) SBA-Pr-Is-TAP.

**Figure 5:**
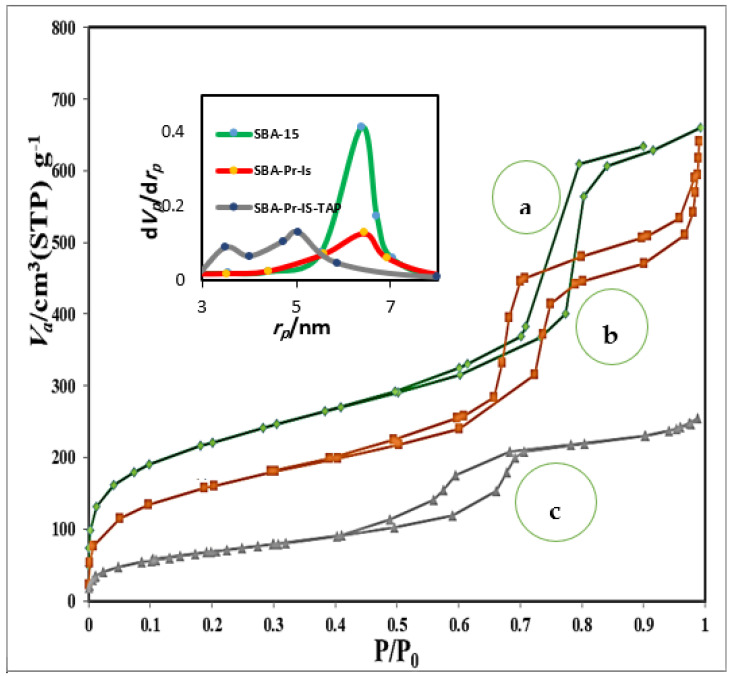
Nitrogen adsorption-desorption isotherms of (**a**) SBA-15, (**b**) SBA-Pr-Is, and (**c**) SBA-Pr-Is-TAP.

SEM and TEM images also proved the nano-ordered arrays of SBA-15 and SBA-Pr-Is-TAP hexagonal nanoporous channels, as shown in Figure 3 and Figure 4. The prepared materials’ physical properties and porosity were investigated by nitrogen adsorption-desorption isotherms (Figure 5). These compounds display type IV isotherms with H1-type hysteresis loops, which proved the pore walls of SBA-Pr-Is and SBA-Pr-Is-TAP are still open. After the attachment of organic compounds on SBA-15, certain changes in physical properties became evident including a reduction in surface area (S_BET_), average pore diameter (d_p_), and pore volume (V_p_) as shown in Table 1. The reasons for the reduced surface area, pore diameter, and pore volume relate to the deterioration of the textural properties of SBA-15 upon the incorporation of the organic moieties (pore diameter and volume) due to partial pore blocking of such organic moieties and surface area due to the partial covering of the surface with the anchored organic moieties.

**Figure 6:**
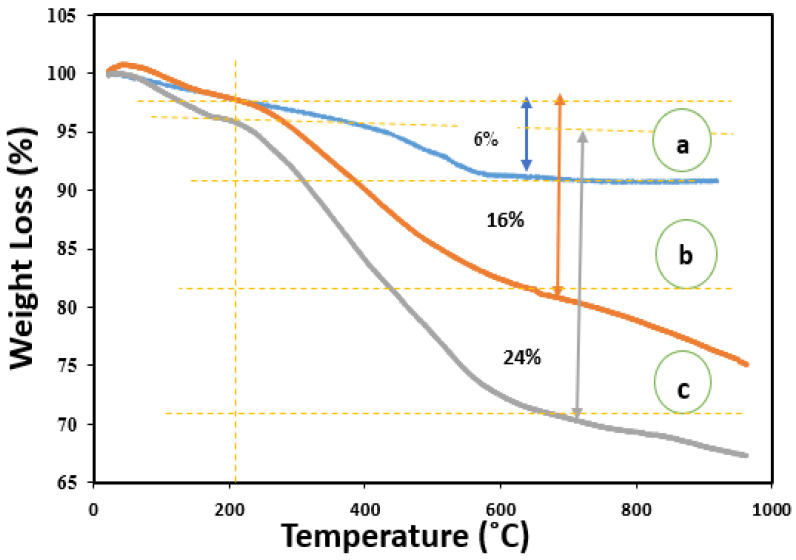
TGA curves of (**a**) SBA-Pr-Cl, (**b**) SBA-Pr-Is, and (**c**) SBA-Pr-Is-TAP.

Thermogravimetry studies confirmed the presence of loaded organic compounds on the SBA-15 surface as shown in Figure 6. In two TGA curves, the weight loss (up to 150 °C) was due to physisorbed water from the mesoporous material. The obtained results pointed out that SBA-Pr-Is-TAP is hydrophilic because of the existence of more nitrogen atoms. The weight loss at 200 and 650 °C was related to the breakdown and removal of organic compounds while that at 700 °C corresponds to the dehydroxylation of silanol groups. TGA curves indicated, respectively, weight losses of ∼6% (0.76 mmol), ∼16% (0.845 mmol), and 24% (0.809 mmol) of organic compounds for SBA-Pr-Cl, SBA-Pr-Is, and SBA-Pr-Is-TAP as expected for materials with increasing loadings of organic content.

## 3. Fluorescence Response of SBA-Pr-Is-TAP to Fe^3+^

**Figure 7:**
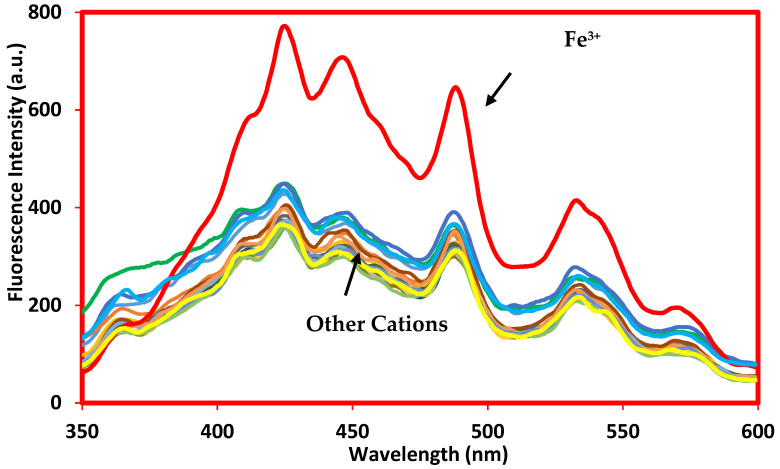
Fluorescence emission of SBA-Is-TAP (3 mL H_2_O suspension, 0.2 g L^−1^) by different cations including Na^+^, Mg^2+^, Al^3+^, K^+^, Ca^2+^, Mn^2+^, Cd^2+^, Co^2+^, Cu^2+^, Li^+^, Zn^2+^, Ni^2+^, Ba^2+^, Hg^2+^, Sr^2+^, Cs^+^, Pb^2+^, Fe^2+^, Cr^3+^, Ag^+^, and Fe^3+^ (100 µL, 1 × 10^−4^ M, λ_ex_ = 220 nm and λ_em_ = 420 nm).

**Scheme 2:**
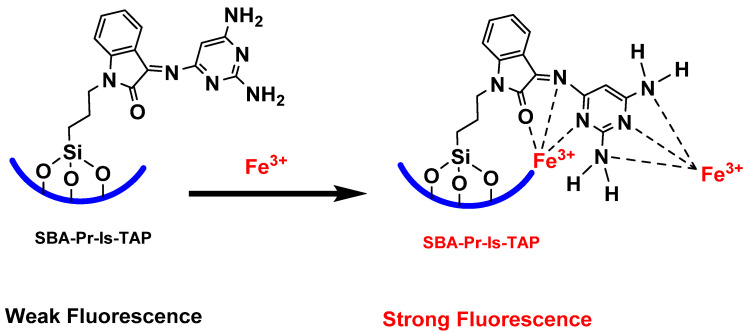
Suggested bonding mode of SBA-Pr-Is-TAP with Fe^3+^.

Fluorescence studies of SBA-Pr-Is-TAP after adding different cations M(NO_3_)_x_ (M = Na^+^, Al^3+^, Cu^2+^, Mg^2+^, K^+^, Cd^2+^, Ca^2+^, Mn^2+^, Co^2+^, Li^+^, Zn^2+^, Ni^2+^, Ba^2+^, Hg^2+^, Sr^2+^, Cs^+^, Pb^2+^, Fe^2+^, Cr^3+^, Ag^+^, and Fe^3+^) were conducted at 420 nm as shown in Figure 7. Luminescence intensities were influenced by adding Fe^3+^ ions as compared to other metal ions, pointing to SBA-Pr-Is-TAP with potential recognition and sensing of Fe^3+^ ions. The interaction between SBA-Pr-Is-TAP and Fe^3+^ was displayed in Scheme 2, believed to be related to the interaction of Fe^3+^ species with nitrogen groups present in the organic moieties of SBA-Pr-Is-TAP.

This high selectivity can be interpreted due to the different charge densities of metal ions and their interaction between guest and host. The charge density (ρ) of a metal ion is defined as the amount of electric charge/unit volume [36,37]. It is one of the most important parameters of the relative electrophilicity of a metal ion for interaction between ligand and ion. The charge densities of metal ions were calculated according to Equation (1):

ρ = q/(4/3 πr^3^) (1)

where q is the formal charge and r denotes the Shannon ionic radius [38]. The calculated charge density of the cations increase in the following order: Al^3+^ (4.67) < Cr^3+^ (3.08) < Fe^3+^ (6.45).

The results indicate that the charge density of Fe^3+^ ion is the largest among all of competition ions. Therefore, the electrophilic ability (capability of forming a coordination complex with an electron-rich ligand) of an Fe^3+^ ion is the strongest among cations. On the other hand, metal ions (as guest) can diffuse into the functionalized nanochannels of SBA-15 (as a host) [38]. The host-guest interaction depends on the concentration of ion and chelating capability into the channels, with Fe^3+^ ions having an enhanced interaction with the strong electrophilicity of amine groups (TAP).

**Figure 8:**
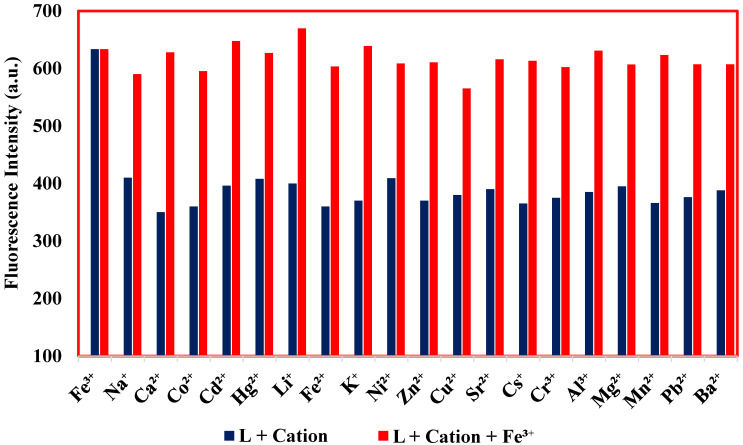
Selectivity of SBA-Pr-Is-TAP (3 mL H_2_O suspension, 0.2 g L^−1^) for Fe^3+^ (100 µL, 1 × 10^−4^ M) using equal amounts of cations (λ_ex_ = 220 nm and λ_em_ = 420 nm).

Selectivity studies of SBA-Pr-Is-TAP towards Fe^3+^ ions were subsequently conducted in the presence of other cations (Figure 8). Spectra were recorded at λ_ex_ = 220 nm and λ_em_ = 420 nm after the addition of SBA-Pr-Is-TAP suspension (0.02 g L^−1^, 3 mL H_2_O) to the 100 µL of Fe^3+^ mixture (1 × 10^−4^ M) with 100 µL (1 × 10^−4^ M) of the mentioned metal cations. Remarkably, results indicated that SBA-Pr-Is-TAP has good selectivity for Fe^3+^ among a range of different tested cations.

**Figure 9:**
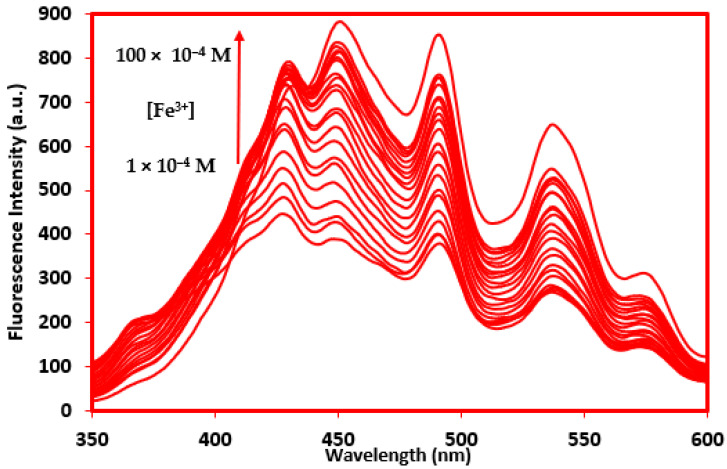
Fluorescence response of SBA-Pr-Is-TAP (3 mL H_2_O suspension, 0.2 g L^−1^) by adding different concentrations of Fe^3+^.

**Figure 10:**
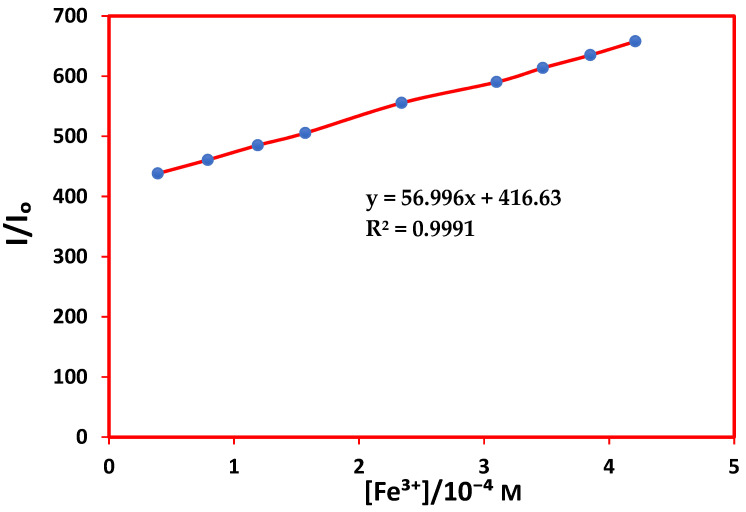
Stern-Volmer plot for titration of SBA-Pr-Is-TAP with different concentrations of Fe^3+^.

A fluorescence titration experiment was also carried out with the addition of various amounts of Fe^3+^ ions (1 × 10^−4^ to 100 × 10^−4^ M) into the aqueous suspension of SBA-Pr-Is-TAP. The relation between fluorescence intensity and different concentrations of Fe^3+^ is shown in Figure 9. Fluorescence intensity was enhanced by increasing Fe^3+^ concentrations. A linear relation between fluorescence intensity and concentration of Fe^3+^ was observed, with R^2^ of 0.9991 as shown in Figure 10. The detection limit was calculated according to the following equation: DL = KS_d_/m. DL was calculated to be 2.43 × 10^−6^, where S_d_ is the standard deviation of the blank solution measured five times, m is the slope of fluorescence intensity of SBA-Pr-Is-TAP versus [Fe^3+^], and K is 3 as a confidence level. It showed good detection of limit in comparison with the other fluorescent compounds in Table 2.

**Figure 11:**
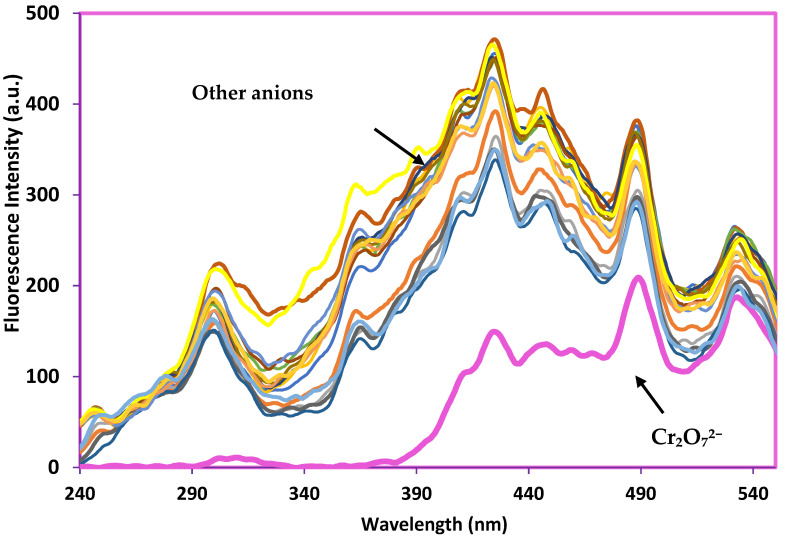
Fluorescence spectra of the aqueous suspended SBA-Pr-Is-TAP (0.2 g L^−1^) in the presence of different anions (100 µL of M^n−^ (1×10^−4^ M)), including I^−^, CN^−^, Cl^−^, F^−^, Br^−^, CO_3_^2−^, SO_4_^2−^, NO_2_^−^, NO_3_^−^, HCO_3_^−^, H_2_PO_4_^−^, CH_3_COO^−^, SCN^−^, S_2_O_3_^2−^, OH^−^, and Cr_2_O_7_^2−^ (λ_ex_ = 220 nm, λ_em_ = 420 nm).

**Scheme 3:**
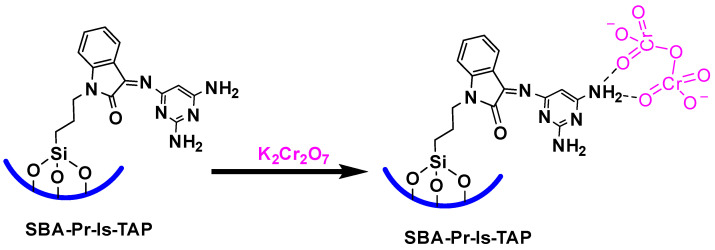
The suggested bonding mode of SBA-Pr-Is-TAP with Cr_2_O_7_^2−^.

Fluorescence studies of SBA-Pr-Is-TAP after adding different anions A^n−^, including = I^−^, CN^−^, Cl^−^, F^−^, Br^−^, CO_3_^2−^, SO_4_^2−^, HCO_3_^−^, H_2_PO_4_^−^, NO_2_^−^, NO_3_^−^, CH_3_COO^−^, SCN^−^, S_2_O_3_^2−^, OH^−^, and Cr_2_O_7_^2−^, was obtained at λ_ex_ = 220 nm, λ_em_ = 420 nm as shown in Figure 11 and Scheme 3. Luminescence intensities were influenced by adding Cr_2_O_7_^2−^ ions as compared to other anions. Therefore, SBA-Pr-Is-TAP also showed a potential recognition and sensing for Cr_2_O_7_^2−^ ions.

**Figure 12:**
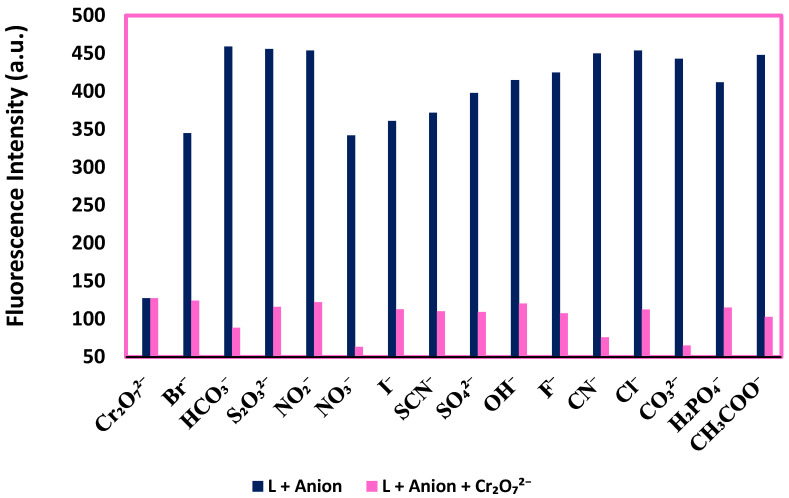
Selectivity of SBA-Pr-Is-TAP (3 mL H_2_O suspension, 0.2 g L^−1^) for Cr_2_O_7_^2−^ (100 µL, 1 × 10^−4^ M) in the presence of equal amounts of anions (λ_ex_ = 255 nm and λ_em_ = 420 nm).

The selectivity of SBA-Pr-Is-TAP towards Cr_2_O_7_^2−^ ions was subsequently investigated in the presence of other anions (Figure 12). In this regard, spectra were recorded at λ_ex_ = 255 nm and λ_em_ = 420 nm after the addition of SBA-Pr-Is-TAP suspension (0.02 g L^−1^, 3 mL H_2_O) to the mixture of Cr_2_O_7_^2−^ (100 µL, 1 × 10^−4^ M) and the mentioned anions (100 µL, 1 × 10^−4^ M). The obtained results showed that the SBA-Pr-Is-TAP has good selectivity for Cr_2_O_7_^2−^.

**Figure 13:**
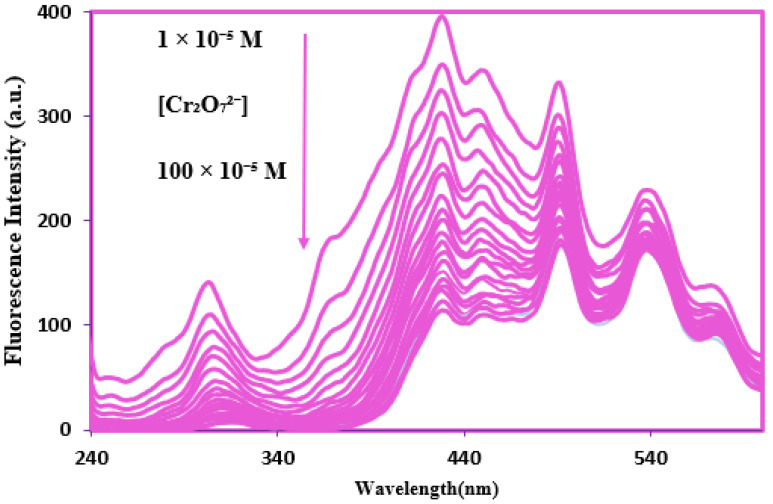
Fluorescence response of SBA-Pr-Is-TAP (3 mL H_2_O suspension, 0.2 g L^−1^) upon the addition of various concentrations of Cr_2_O_7_^2−^.

**Figure 14:**
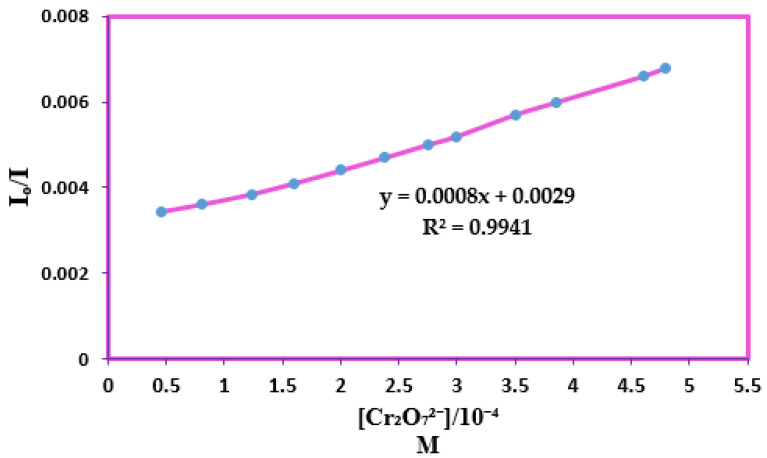
Stern-Volmer plot for titration of SBA-Pr-Is-TAP with different concentrations of Cr_2_O_7_^2−^.

A fluorescence titration experiment was next carried out with the addition of various amounts of Cr_2_O_7_^2−^ ions (1 × 10^−5^ to 100 × 10^−5^ M) into the aqueous suspension of SBA-Pr-Is-TAP (3 mL H_2_O suspension, 0.2 g L^−1^). The relation between fluorescence intensity and different concentrations of Cr_2_O_7_^2−^ is shown in Figure 13. Fluorescence intensity decreased while the concentration of Cr_2_O_7_^2−^ ions gradually increased. A linear relation between fluorescence intensity and concentration of Cr_2_O_7_^2−^ was found, with R^2^ of 0.9941 as shown in Figure 14. The detection limit was calculated (similarly to that for Fe^3+^) to be 3.96 × 10^−7^ M and compared with the other chemosensor compounds in Table 3.

The quenching mechanism of SBA-Pr-Is-TAP with Cr_2_O_7_^2−^ was subsequently investigated using the Stern-Volmer equation. Figure 14 shows a linear plot of I_0_/I against the concentration of Cr_2_O_7_^2−^ ions which indicates a static quench. UV-Vis spectrum of Cr_2_O_7_^2−^ ions exhibits two maximum absorption wavelengths at 257 and 350 nm in UV range and another at 440 nm in the visible domain which has good spectral overlap with the fluorescence emission peak of SBA-Pr-Is-TAP [47]. Therefore, the strong inner filter effect (IFE) possibly occurs after the addition of Cr_2_O_7_^2−^ in the dispersed SBA-Pr-Is-TAP [48,49]. In several reports, IFE has been proposed as a fluorescence quenching mechanism in optical dichromate sensors [18,50]. This quenching mechanism was proposed based on the formation of hydrogen bonding between the oxygen group of Cr_2_O_7_^2−^ and hydrogen atoms belong to the amine or carboxyl group of functionalized TAP into the nanochannel of SBA-15 [51]. This hydrogen bonding may change the electronic distribution of TAP; thereby, the fluorescence emissions can be quenched and intensity changed. Therefore, besides IFE, hydrogen bonding is suggested as fluorescence quenching mechanism of SBA-Pr-Is-TAP with Cr_2_O_7_^2−^.

## 4. Conclusions

SBA-Pr-Is-TAP as organic-inorganic nanohybrid via SBA-15 modification with isatin and 2,4,6-triaminopyrimidine was synthesized and characterized. Textural and physicochemical properties confirmed the successful incorporation of organic groups on SBA-15. Fluorescence studies demonstrated the hybrid or an organic material can be a selective sensor for Fe^3+^ and Cr_2_O_7_^2−^ recognition in H_2_O, with detection limits of 2.43 × 10^−6^ and 3.96 × 10^−7^ M, respectively.

## 5. Experimental Section

### 5.1. Synthesis of SBA-15, SBA-Pr-Cl, and SBA-Pr-Is

Materials were prepared based on our previous reported contributions [27,31,33].

### 5.2. Synthesis of SBA-Pr-Is-TAP

SBA-15-Pr-Is (1 g) was dispersed in dry EtOH. 2,4,6-triaminopyrimidine (0.62 g, 5 mmol) was added to the mixture of reactions. It was refluxed for 24 h, and then filtered to obtain the dark brown powder, which was soxhlated with dried EtOH to obtain pure SBA-15-Pr-Is.

Low-angle X-ray scattering measurements were performed using an X’Pert Pro MPD diffractometer using Cu K_α_ radiation (λ = 1.5418 Ǻ).

N2 adsorption-desorption isotherms were obtained using a BELSORP-miniII instrument at liquid nitrogen temperature (−196 °C). Degassing process for all samples was performed at 100 °C before the measurements. The Brunauer-Emmett-Teller (BET) and Barrett-Joyner-Halenda (BJH) equations were applied on sorption data using BELSORP analysis software to calculate the physical properties of materials such as the specific surface area, pore diameter, pore-volume, and pore size distribution.

Thermogravimetric analyses (TGA) were carried out by a TGA Q50 V6.3 Build 189 instrument from ambient temperature to 1000 °C with a ramp rate of 10 ℃ min^−1^ in the air. SEM analysis was performed on a Hitachi S-4160 operated at 30 kV.

FT-IR spectra were obtained in KBr disks on a RAYLEIGH WQF-510A FT-IR spectrometer in the 600–4000 cm^−1^ region. Fluorescence measurements were collected on a Cary Eclipse Fluorescent Spectrophotometer.

Additional information can be found in the Supplementary Materials submitted with this article.

## 6. Calculation of Detection Limit (DL)

DLs can be calculated based on the standard deviation of the response (S_d_) of the curve and the slope of the calibration curve (m) at levels approximating the DL according to the formula: DL = 3 (S_d_/m). The standard deviation of the response can be determined based on the standard deviation of y-intercepts of regression lines. The slope and S_d_ can be obtained with one order of magnitude of the calibration curve.

## Figures and Tables

**Table 1:** Structural parameters of SBA-15, SBA-Pr-Is, and SBA-Pr-Is-TAP.

Sample	S_BET_ (m^2^·g^−1^)	V (cm^3^·g^−1^)	D_BJH_ (nm)
SBA-15	672	0.69	4.6
SBA-Pr-Is	550	0.64	4.1
SBA-Pr-Is-TAP	250	0.37	0.8

**Table 2:** Comparison of Detection Limits of different fluorescent compounds.

Compound	Ion	DL
Carbon quantum dot [39]	Fe^3+^	2.78 × 10^−6^ M
SBA-15-DNPH [40]	Fe^3+^	29 × 10^−6^ M
1,4-Dihydropyridine Colorimetric [41]	Fe^3+^	8.3 × 10^−6^ M
A pyrene-based dual chemosensor [42]	Fe^3+^	2.0 × 10^−6^ M

**Table 3:** Comparison of Detection Limits of different fluorescent compounds.

Compound	Ion	DL
Mitochondria-targeted acridine-based [43]	Cr_2_O_7_^2−^	1.6 × 10^−7^ M
{[Cd(TCPA)(L)]·H_2_O}_n_ [44]	Cr_2_O_7_^2−^	4 × 10^−7^ M
Zn^II^-MOF [45]	Cr_2_O_7_^2−^	3 × 10^−7^ M
GSH@CDs-Cu NCs [46]	Cr_2_O_7_^2−^	9 × 10^−7^ M

## Data Availability

Not applicable.

## Supplementary Materials

Supplementary materials are available online at https://www.mdpi.com/article/10.3390/nano11102533/s1.
