# Supplementary material for: SBA-Pr-Is-TAP Functionalized Nanostructured Silica as a Highly Selective Fluorescent Chemosensor for Fe3+ and Cr2O72− Ions in Aqueous Media
Source: Nanomaterials (Basel). 2021 Sep 28;11(10):2533. doi: 10.3390/nano11102533 (PMC8537779; doi:10.3390/nano11102533)
Supplement: Supplementary file 1 [file nanomaterials-11-02533-s001.zip › nanomaterials-1268506-supplementary.pdf]

nanomaterials

MDPI

Supporting information

SBA-Pr-Is-TAP Functionalized Nanostructured Silica as a Highly Selective Fluorescent Chemosensor for Fe^{3+} and $Cr_2O_7^{2-}$ Ions in Aqueous Media

Ghodsi Mohammadi Ziarani [1,*], Maryam Akhgar [1], Fatemeh Mohajer [1], Alireza Badiei [2] and Rafael Luque [3,*]

[1] Department of Chemistry, Faculty of Physics and Chemistry, Alzahra University, Tehran 1993893979, Iran; m.akhgar@student.alzahra.ac.ir (M.A.); f.mohajer@alzahra.ac.ir (F.M.)
[2] School of Chemistry, College of Science, University of Tehran, Tehran 1417614411, Iran; abadiei@khayam.ut.ac.ir
[3] Departamento de Quimica Organica, Campus de Rabanales, Universidad de Cordoba, Edificio Marie Curie, 14014 Córdoba, Spain
* Correspondence: gmohammadi@alzahra.ac.ir (G.M.Z.); rafael.luque@uco.es (R.L.); Tel./Fax: +98-21-8861-3937 (G.M.Z.)

Experimental Section

Materials and Physical Measurements

2,4,6-tri-aminoprymidine, isatin, (3-chlorpropyl)triethoxysilane (Sigma Aldrich), tetraethylorthosilicate (TEOS), triethylamine, potassium carbonate, ethanol, and toluene (Merck). All chemicals and solvents were used without further purification. Low-angle XRD methods were recorded by using a X'Pert Pro MPD using Cu-Kα radiation (λ=1.5405 Å). Brunauer–Emmett–Teller (BET) procedures were performed by using a Belsorp, mini II Japan. Thermogravimetric analyzes (TGA) were measured by a TGA Q50 V6.3 with a rising rate of 10 °C min^{-1} in the air. Scanning electron microscopy (SEM) analysis was recorded on Hitachi S-4160. FT-IR spectra were obtained from the KBr disc using a FT-IR Bruker Tensor 27 tool. Fluorescence diagrams were composed with a Cary Eclipse Fluorescent Spectrophotometer. TEM analysis was recorded from ZEISS company which was made in Germany, EM10C-100 KV moldel.

Synthesis of SBA-15

The SBA-15 was prepared based on the previous reports.1

Synthesis of SBA-Pr-Cl

The synthesis process for the functionalization of the SBA-15 with the chlorpropyl moiety was prepared according to an informed procedure [2]. 1 g of dried and calcined SBA-15 was dispersed in 50 mL dried toluene with stirring. Then, 5 mmol (3-chlorpropyl) triethoxysilane was slowly added to the homogenous solution and was refluxed for 24 h. Lastly, the solid product was filtered, washed with ethanol, and dried at room temperature.

Synthesis of SBA-Pr-Is

SBA-Pr-Is was obtained according to an informed procedure before 3. Isatin (5 mmol, 0.73 g) and triethylamine (10 mmol) were added to a suspension of 1 g of the prepared SBA-Pr-Cl in dried toluene. Then the homogenous solution was refluxed for 24 h. the final product was washed several times with dried ethanol and dried at room temperature.

References

1. Zhao, D.; Feng, J.; Huo, Q.; Melosh, N.; Fredrickson, G.H.; Chmelka, B.F.; Stucky, G.D. *Science.* 1998, 279, 548–552. https://doi.org/10.1126/science.279.5350.548
2. Karimi, M.; Badiei, A.; Mohammadi Ziarani, G. *Inorganica Chim. Acta* **2016**, *450*, 346–352. https://doi.org/10.1016/j.ica.2016.06.026
3. Lashgari, N.; Badiei, A.; Mohammadi Ziarani, G. *J Phys Chem Solids* **2017**, *103*, 238–248. https://doi.org/10.1016/j.jpcs.2016.11.021
